# The impact of demographic and risk factor changes on coronary heart disease deaths in Beijing, 1999–2010

**DOI:** 10.1186/1471-2458-9-30

**Published:** 2009-01-22

**Authors:** Jun Cheng, Dong Zhao, Zhechun Zeng, Julia Alison Critchley, Jing Liu, Wei Wang, Jiayi Sun, Simon Capewell

**Affiliations:** 1 Department of Epidemiology, Beijing Institute of Heart, Lung & Blood Vessel Diseases, Beijing Anzhen Hospital affiliated to the Capital Medical University, Beijing, PR China; 2 Institute of Health and Society, Newcastle University, Newcastle, UK; 3 The Division of Public Health, University of Liverpool, Liverpool, UK

## Abstract

**Background:**

Recent, dramatic increases in coronary heart disease (CHD) mortality in China can be mostly explained by adverse changes in major cardiovascular risk factors. Our study aimed to assess the potential impact of subsequent changes in risk factors and population ageing on CHD deaths in Beijing between 1999 and 2010.

**Methods:**

The previously validated IMPACT model was used to estimate the CHD deaths expected in 2010, with treatment uptakes being held constant at levels measured in 1999, comparing three scenarios: a) taking into account the ageing of the population but assuming no further changes in major risk factor levels from 1999 or, b) if recent risk factor trends continued until 2010 or, c) if there was a 0.5% annual reduction in each risk factor.

**Results:**

Population ageing alone would result in approximately 1990 additional deaths in 2010 compared with 1999, representing an increase of 27%. Continuation of current risk factor trends would result in approximately 3,015 extra deaths in 2010, [a 40% increase]; three quarters of this increase would be attributable to rises in total cholesterol levels. Thus, demographic changes and worsening risk factors would together result in a 67% increase in CHD deaths. Conversely, assumed 0.5% annual reductions in risk factors (a mean population level decline of 0.3 mmol/L for total cholesterol in both genders, and smoking prevalence declining by 3.0% for men and 4.1% for women, body mass index by 1.3 kg/m^2 ^for men and 1.4 kg/m^2 ^for women, diabetes prevalence by 0.4% in both genders, and diastolic blood pressure by 4.7 mmHg for men and 4.4 mmHg for women) would result in some 3,730 fewer deaths, representing a 23% decrease overall. These findings remained consistent in sensitivity analyses.

**Conclusion:**

CHD death rates are continuing to rise in Beijing. This reflects worsening risk factor levels, compounded by demographic trends. However, the adverse impact of population ageing on CHD burden could be completely offset by eminently feasible improvements in diet and smoking.

## Background

Coronary heart disease (CHD) mortality rates have halved in most industrialized countries since the 1980s [1]. However, most developing countries, including China, have experienced substantial increases in CHD mortality in recent decades [2]. CHD is thus projected to be the leading global cause of death and disability by 2020 [3].

A variety of CHD policy models have therefore been developed to help explain the observed changes in CHD mortality in terms of changes in cardiovascular risk factors and treatments [4-6]. Analyses conducted in different countries have consistently suggested that reductions in cardiovascular risk factors account for larger CHD mortality effects than treatments [7-14]. Using these models to explain future trends in CHD mortality in terms of risk factor trends, or demographic change, can be potentially helpful for policy makers. Such analyses can also help identify the most effective and cost-effective prevention strategies, especially for economically developing countries.

The IMPACT Model was developed by Capewell and colleagues and successfully used to help explain the observed declines in CHD mortality resulting from improving risk factor levels and cardiological treatments in many developed countries, including the UK, the USA, New Zealand and Finland [9-13,15], and to estimate the number of additional deaths that could be prevented or postponed due to further declines in risk factors [16]. Subsequently, the model was used to evaluate potential future options [9-13,17]. More recently, the IMPACT model has also been applied to populations where CHD mortality rates are increasing [18]. In Beijing between 1984 and 1999, the CHD mortality rates rose by 50% in men and 27% in women aged 35–74 years, and most of these increases could be attributed to substantial increases in total cholesterol levels (mean population level rose by 1.1 mmol/L) [18].

In this paper, we now extend this Beijing population model from 1999 to estimate the number of CHD deaths anticipated in Beijing in 2010 under three contrasting scenarios: a) assuming simple population ageing, b) assuming that recent adverse trends in risk factors continue to worsen, and c) more optimistically assuming 0.5% annual reductions in major cardiovascular risk factors, as already achieved in many other populations [17]. These estimates might provide a potentially useful reference to inform the planning of future CHD prevention and treatment strategies in Beijing.

## Methods

### Identification of relevant data

The model includes information on mortality and risk factor trends in the Beijing population aged 35–74 years. Data on the population size and gender- and age-specific CHD mortality were obtained from the fifth National Population Census (2000) and Beijing Municipal Bureau of Statistics [19]. The number of CHD deaths in 1999 was therefore calculated by applying the mortality and population size by gender and age group for Beijing. The projected population estimates in 2010 were provided by the Chinese Statistics Association. The changes in population size between 1999 and 2010 are listed in Table 1. Five independent cardiovascular risk factor surveys were carried out in the stratified-random sampling subjects from MONICA monitoring population in Beijing from 1984–1999. Trends of major cardiovascular risk factors were compared, including systolic and diastolic blood pressure, prevalence of hypertension, serum total cholesterol, glucose, body mass index and smoking prevalence. Population risk factor trend data came from these quality-assured MONICA and Sino-MONICA studies [20-22].

**Table 1:** Gender- and age-specific population sizes in Beijing in 1999 and projections to 2010

**Groups**	**1999**	**2010**	***% population increase***
**Men**			

**35–44**	1,402,305	1,330,195	*-5.1*

**45–54**	899,529	1,351,654	*50.3*

**55–64**	506,847	841,221	*66.0*

**65–74**	410,002	426,058	*3.9*

**Total men**	**3,218,683**	**3,949,128**	***22.7***

			

**Women**			

**35–44**	1,249,534	1,134,015	*-9.2*

**45–54**	883,857	1,258,104	*42.3*

**55–64**	557,514	875,272	*57.0*

**65–74**	412,808	499,409	*21.0*

**Total women**	**3,103,715**	**3,766,800**	***21.4***

			

**Men & Women**	**6,322,396**	**7,715,928**	***22.0***

This IMPACT Model uses two validated methods to describe the relationships between population changes in specific cardiovascular risk factors and the consequent changes in population CHD mortality rates [5,11,18]. The first is a regression method. (More detailed information is provided in Additional file 1).

1) Regression coefficients from meta-analyses of large cohort studies were used to assess the mortality effects of changes in cholesterol [23,24], body mass index (BMI) [25] and diastolic blood pressure [26]. The subsequent additional deaths, stratified by age and gender, were calculated as the product of the number of CHD deaths observed in 1999 (the base year), the absolute risk factor change and the regression coefficient.

2) Alternatively, another separate method was used in the absence of suitable β-coefficient, and also as an independent validation. Population attributable risk fractions (PARF) were used to assess the effects of changes in smoking and diabetes prevalence. The mortality outputs were estimated as the product of the CHD deaths in the base year (1999), multiplied by the increase in PARF between 1999 and 2010. The mortality changes expected from the trends in each risk factor were obtained from Chinese cohort studies (smoking [27,28]) and meta-analyses of large cohort studies (diabetes [29,30]). Since there have been substantial recent changes in both the level and prevalence of smoking in China, the impact of smoking was modeled as three categories based on cigarette consumption (cigarettes/day: 1–9, 10–19, and ≥ 20).

The β-coefficients and relative risk were derived from studies in America, Europe and Asia, and more than ten cohort studies conducted in mainland China. The number of participants ranged from 44 to 49,000, and 1%–64% were women. The age range of participants was 35–89 years old, and the duration of follow-up was from 3.0 to 21.2 years (see Additional file 1).

### Estimating the number of CHD deaths in 2010

#### Scenario 1: Simply assuming population ageing between 1999 and 2010

The deaths expected in 2010 were calculated by indirect age standardization, applying the age-specific death rates in 1999 to the 2010 projected population. This therefore assumed no change in age and gender-specific CHD mortality rates between 1999 and 2010.

#### Scenario 2: Continuation in recent risk factor trends between 1999 and 2010

All the major risk factors (smoking prevalence, total cholesterol level and diastolic blood pressure) showed upward trends for both genders from 1984 to 1999, except for smoking prevalence in women, as measured in quality-assured MONICA and Sino-MONICA studies, and serial national surveys [20-22]. In this scenario, we therefore assumed that the recent trends in risk factor levels observed in Beijing population between 1984–1999 simply continued at the same relative rates until 2010. In the absence of detailed trend data in the elderly, we conservatively assumed that the risk factor changes in the older population aged 65–74 years rose at only half the rate of the increases seen in younger age groups [17]. The observed risk factor levels in 1999 and those projected in 2010 among subjects aged 35–64 years are detailed in Table 2.

**Table 2:** Risk factor levels at 1999 baseline and projections to 2010 for men and women aged 35–64 years*

**Risk factors**	**1999 (baseline)**		**2010 (assuming current trends continue)**		**2010 (assuming 0.5% annual reductions)**	
	***men***	***women***	***men***	***women***	***men***	***women***

**Cholesterol **(mmol/L)	5.3	*5.3*	5.8	*5.9*	5.0	*5.0*

**Smoking **(prevalence)	58.0%	*8.9%*	68.6%	*4.8%*	52.0%	*4.8%*†

**BMI (kg/m**^2^**)**	25.1	*25.1*	26.8	*25.1*	23.8	*23.7*

**Diabetes **(prevalence)	7.1%	*7.2%*	17.2%	*14.4%*	6.7%	*6.8%*

**Diastolic blood pressure **(mmHg)	85.9	*80.9*	86.3	*81.7*	81.2	*76.5*

BMI indicates body mass index

* Risk factor changes in the older population aged 65–74 years, where data was generally lacking, were conservatively assumed to rise at half the rate of the increases seen in younger age groups *(data for this age group not shown in table)*.

† Recent trends already represent an annual fall of approximately 0.5%.

#### Scenario 3: Optimistically assuming 0.5% annual risk factor reductions between 1999 and 2010

Similar calculations were then performed, more optimistically assuming 0.5% annual reductions in cholesterol, diastolic blood pressure, BMI and diabetes prevalence in both genders. Similar rates of reduction have been achieved in other rapidly industrializing populations, e.g. Poland, the Czech Republic and Hungary [31]. Smoking prevalence has already fallen in women in Beijing, and it also now appears to be falling nationally in men.

### Treatment trends

All medical and surgical treatment uptakes in the present study were held constant at levels measured in 1999, reflecting current resource constraints. These levels, and the data sources on which they were based, have been described previously [18].

### Sensitivity analysis

Most model parameters were subject to some uncertainty, with potential overestimation or underestimation of benefit. Sensitivity analyses were therefore performed using the analysis of extremes method [5,11]. In addition to the 'best' estimate of mortality change, minimum and maximum estimates were also generated using the highest and lowest plausible values for each risk factor.

### Study ethics

This study design and investigation were ethically approved by the ethical committee of Beijing Anzhen Hospital affiliated to the Capital Medical University.

## Results

### Changes in the population structure between 1999 and 2010

The gender- and age-specific characteristics of the population in the base year and final year are summarized in Table 1. The population size will increase by approximately 23% for men and 22% for women aged 35–74 years. The estimated age-specific percentage increases in the age groups of 35–44, 45–54, 55–64 and 65–74 years between 1999 and 2010 would be -5%, 50%, 66% and 4%, respectively, in men, and -10%, 43%, 57% and 21%, respectively, in women, reflecting current demographic changes in the Beijing population.

### Estimated CHD deaths in 2010

#### Scenario 1: Simply due to demographic changes

Simply assuming that the age-specific death rates in 1999 continue to apply to the older population in 2010, approximately 9,390 CHD deaths would be expected in 2010 (5,565 among men and 3,825 in women, representing overall increases of 24.2% and 31.0% from 1999, respectively).

#### Scenario 2: Based on the current risk factor trends

**Figure 1:**
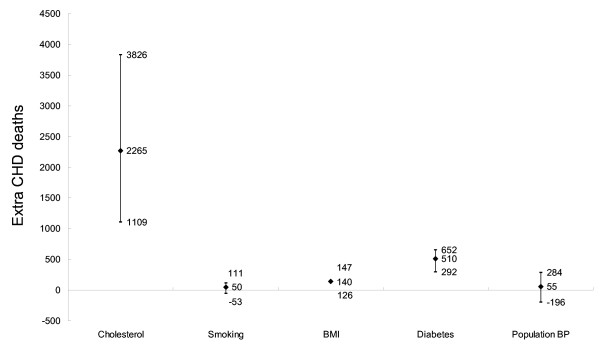
**CHD mortality trends in Beijing 1999–2010: extra deaths attributable to increases in risk factors: results of a sensitivity analysis**.

A projection of the recent trends indicated that risk factors would continue to increase from 1999, as detailed in Table 2. This would lead to approximately 3,015 extra CHD deaths in 2010, *(minimum estimate 1,922, maximum estimate 4,376*, Figure 1. *Black diamond indicates the best estimate, and short bar the minimum and maximum estimates)*. These 3,015 additional deaths would result from approximately 2,265 (75%) attributable to increases in total cholesterol level (from 5.3 mmol/L to 5.8 mmol/L), approximately 510 attributable to an increased prevalence of diabetes (from 7.1% to 15.8%), approximately 140 attributable to increase in BMI (from 25.1 to 26.0), approximately 55 additional deaths attributable to a slight increase in diastolic blood pressure (from 83.5 mmHg to 84.1 mmHg), and approximately 50 attributable to an overall increase in smoking prevalence in men (from 58.0% to 68.6%) but a decrease in women (from 8.9% to 4.8%).

#### Scenario 3: Optimistic assumption: small reductions in risk factors

**Figure 2:**
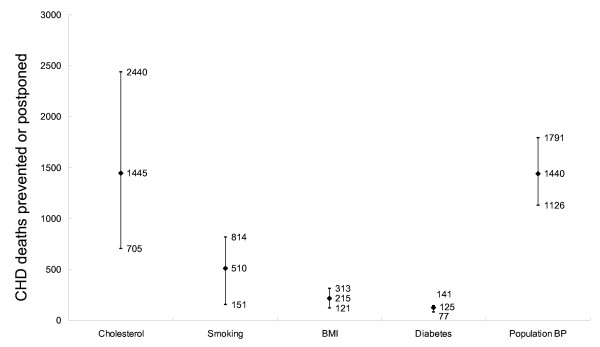
**CHD mortality trends in Beijing 1999–2010: deaths prevented or postponed by reductions in risk factors: results of a sensitivity analysis**.

A total of approximately 3,730 CHD deaths *(minimum estimate 2,180, maximum estimate 5,499) *may be prevented or postponed by modest but feasible reductions in each major risk factor. The biggest benefits would come from decreases in the total cholesterol levels, followed by decreases in the mean population diastolic blood pressure and smoking rates (Figure 2. *Black diamond indicates the best estimate, and short bar the minimum and maximum estimates*). The specific results are detailed below.

First, approximately 1,445 fewer deaths *(minimum 705, maximum 2,440) *if the mean population **cholesterol **levels fell from 5.3 mmol/L to 5.0 mmol/L in both men and women.

Second, approximately 510 fewer deaths *(minimum 151, maximum 814) *if the **smoking **rates fell from 58.0% to 52.0% in men and from 8.9% to 4.8% in women.

Third, approximately 215 fewer deaths *(minimum 121, maximum 313) *if the mean population **BMI **fell from 25.1 kg/m^2 ^to 23.8 kg/m^2 ^in men and from 25.1 kg/m^2 ^to 23.7 kg/m^2 ^in women.

Fourth, approximately 125 fewer deaths *(minimum 77, maximum 141) *if the population **diabetes **prevalence fell from 7.1% to 6.7% in men and from 7.2% to 6.8% in women.

Fifth, approximately 1,440 fewer deaths *(minimum 1,126, maximum 1,791) *if the mean population diastolic **blood pressure **level fell from 85.9 mmHg to 81.2 mmHg in men and from 80.9 mmHg to 76.5 mmHg in women.

### Mortality changes by age and gender

**Figure 3:**
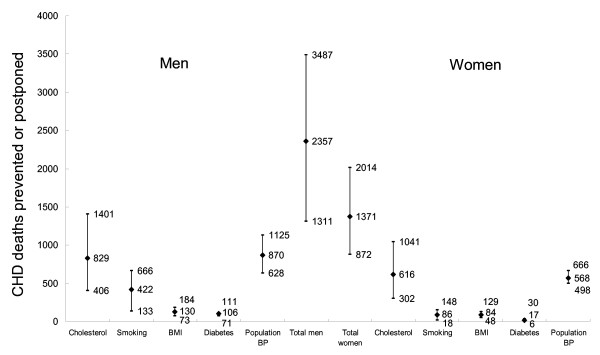
**Contribution of 0.5% annual reductions in risk factors to the projected CHD death reduction in Beijing by gender, 1999 to 2010: results of a sensitivity analysis**.

Changes in population-based mortality rates during 1999–2010 under the three contrasting scenarios are detailed in Table 3. Overall, a larger mortality decrease would be seen in men (approximately 2,355 DPPs) than in women (approximately 1,370 DPPs). In women, the biggest benefits would come from improved cholesterol levels, followed by improved diastolic blood pressure and a decreased smoking rate. Whereas in men, the biggest benefits would come from improved diastolic blood pressure and cholesterol level, followed by improvements in smoking rates (Figure 3. *Black diamond indicates the best estimate, and short bar the minimum and maximum estimates*).

More benefits would be gained in the youngest age groups in both genders (Table 3).

**Table 3:** Gender- and age-specific changes in population-based mortality rates during 1999–2010 with current risk factor trends or 0.5% annual reductions in each risk factor

**Age groups**	**Men**		**Women**	
	
	**Death rates(1/100000)**	***% change (%) (1999–2010)***	**Death rates(1/100000)**	**% change (%) (1999–2010)**
***35–44 years***				

**Rate in 1999**	**31.6**	**-**	**7.9**	**-**

**i) Current trend**	40.4	*27.8*	7.8	*-1.3*

**ii) 0.5% reduction**	14.7	*-53.5*	3.9	*-50.6*

***45–54 years***				

**Rate in 1999**	**71.1**	**-**	**27.0**	**-**

**i) Current trend**	103.8	*46.0*	38.9	*44.1*

**ii) 0.5% reduction**	43.3	*-39.1*	18.0	*-33.3*

***55–64 years***				

**Rate in 1999**	**207.2**	**-**	**135.1**	**-**

**i) Current trend**	293.4	*41.6*	204.5	*51.4*

**ii) 0.5% reduction**	137.2	*-33.8*	92.5	*-31.5*

**65–74 years**				

**Rate in 1999**	**572.2**	**-**	**442.6**	**-**

**i) Current trend**	601.6	*5.1*	613.7	*39.6*

**ii) 0.5% reduction**	298.8	*-47.8*	274.9	*-37.9*

				

***TOTAL (all ages)***				

**Rate in 1999**	**139.2**	**-**	**94.0**	**-**

**i) Current trend**	176.5	*26.8*	144.2	*53.4*

**ii) 0.5% reduction**	81.2	*-41.7*	65.1	*-30.7*

Positive value indicates an increase in mortality, and negative value a decrease.

### Sensitivity analyses

**Figure 4:**
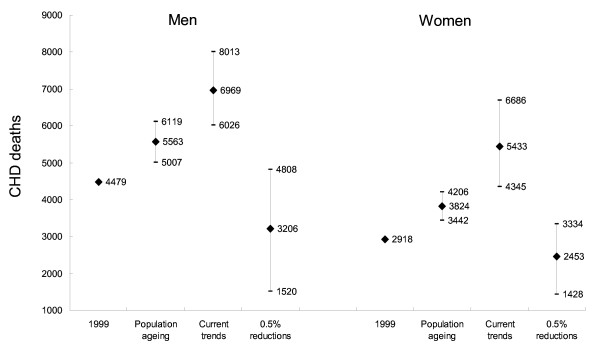
**Summary of observed CHD deaths in 1999 and projections to 2010 under the three contrasting scenarios: results of a sensitivity analysis**.

The principal results remained stable in the sensitivity analysis. (Figures 1, 2, 3, 4. *Black diamond indicates the best estimate, and short bar the minimum and maximum estimates*). Figure 3 shows the sensitivity analysis for Scenario 3. Thus, irrespective of whether best, minimum or maximum estimates were considered, modest reductions in cholesterol were likely to decrease mortality more than comparable reductions in smoking, diabetes or obesity (Figure 3).

## Discussion

This study analyzed current trends in risk factor levels and population ageing to estimate the number of CHD deaths expected in the Beijing population by 2010. Compared with 1999, approximately 1,990 additional deaths might be expected simply due to population ageing, representing a 27% increase. Most worryingly, if recent adverse trends in major risk factors also continue, Beijing will experience over 12,000 CHD deaths in 2010, representing a 67% increase. Alternatively, if modest reductions in major risk factors (0.5% per year) were achieved, as in many other countries, these reductions would decrease CHD deaths by approximately 23%, in spite of population ageing (Figure 2).

The impact of population ageing on CHD mortality was very obvious in this study. In 2010, a substantial increase in older people (about 1.4 million more citizens aged 35–74 years) is expected in Beijing, representing an increase of approximately 22%. Even if the risk factor profiles and treatments remain as good as they were in 1999, these demographic trends mean that the projected CHD deaths in 2010 will rise above 9,300, representing a 27% increase compared with 1999. The number of elderly women is projected to increase far more substantially than the number of elderly men, resulting in a particularly large mortality burden in women. This gender difference is partly explained by a differential mortality in men from competing causes at earlier ages (mainly due to their very high smoking prevalence) [32,33]. Conversely, CHD deaths in the youngest age groups will decrease due to their decline in population size (attributable to a reduction in the birth rate since 1975) [34].

Although several studies have examined the decline in CHD mortality in Western countries [7,9-13], very few studies have been conducted in developing countries or in Asia [18,35,36]. Beijing has witnessed a dramatic increase in CHD mortality during recent decades, much of which can be attributed to the rising population cholesterol levels [18]. Furthermore, the incidence rate of CHD showed a corresponding upward trend in Beijing during 1987 to 1993 [20]. Similarly, the CHD epidemic in Singapore can be largely explained by higher cholesterol levels [35]. These trends are consistent with the observed changes from traditional to Western diet [37], with a higher consumption of dietary saturated fat and lower consumption of polyunsaturated fats, vegetables and carbohydrates [35]. If the recently observed adverse trends in risk factors levels continue in Beijing, there would be 3,000 more deaths annually by 2010 than otherwise expected based on demographic trends (population ageing) alone. Impressively, approximately three-quarters of this increase would reflect increases in total cholesterol levels. In the mid-1980s, cholesterol and obesity levels in Beijing were the lowest among all the MONICA populations [38]. However, the subsequent massive increases in cardiovascular risk factor levels, especially cholesterol, have resulted in many additional CHD deaths because cholesterol is a particularly powerful CHD risk factor [18,38]. Intriguingly, in Japan, decreasing CHD mortality rates have been reported despite modest increases in the total cholesterol levels and similarly high smoking rates to Beijing. However, the high consumption of cardioprotective oily fish in Japan may have helped to offset the unfavourable effects of other cardiovascular risk factors [36]. In Beijing, the overall population diastolic blood pressure level increased slightly and made only a small contribution to the predicted change in CHD mortality. Improvements in hypertension treatment may have contributed [18,39].

Modest reductions in major risk factors could hugely affect the number of future CHD deaths in Beijing, despite population ageing. Annual 0.5% reductions in major risk factors could result in some 3,730 fewer deaths in 2010, representing a 23% decrease compared with 1999. Effective strategies targeting modifiable risk factors could therefore partly offset the adverse impact of unmodifiable risk factors, the most powerful being age.

Small but powerful risk factor reductions have actually been observed elsewhere [17]. Similarly modest falls in the smoking rate, cholesterol level and blood pressure have all been achieved in other industrializing populations, such as Poland and the Czech Republic [31], and thus appear to be feasible in Beijing. Encouragingly, a citywide public smoking ban was enacted in Beijing in 1996, and a nationwide survey confirmed that the smoking rates in China have fallen in recent years [40]. The signing of the Framework Convention on Tobacco Control by the government of China will further promote reductions in smoking. However, reductions in obesity and diabetes appear more challenging to achieve, with adverse trends continuing in the USA and Western Europe [41]. Furthermore, even if the proposed modest reductions in smoking and blood pressure were actually achieved by 2010, the levels in Beijing would remain higher than in most other countries [12,17,38,42]. There is clearly a need for further, more substantial reductions in smoking and blood pressure levels. Such declines would be expected to greatly reduce the burden of CHD [43].

This modeling work can provide useful information, promote evidence-based health policy making, and improve the effectiveness of prevention strategies. Our study results showed that there is substantial potential benefit from reductions in major cardiovascular risk factors by modifying lifestyle, even with the growing and ageing population. The achievable interventions targeting these major risk factors could produce substantial declines of coronary mortality, as already achieved elsewhere [44].

Like any forecasting tool, this study has a number of strengths including the use of a transparent modelling methodology previously replicated in a range of populations. It also contains a number of limitations. Firstly, it considers only mortality, but not years of life lost or morbidity. Life expectancy or life-years gained should be included in future versions of this model [43,45,46]. Secondly, potential interactions among risk factors and potential lag effects were not considered in this comparatively simple, cell-based model. Thus, four important assumptions were made in the model. Firstly, that by using independent coefficients from multivariate analyses, reducing one risk factor would not affect another one. In reality, multiplicative interactions between various risk factors may further decrease the CHD risk [47]. Secondly, assuming an immediate CHD mortality benefit from risk factor modifications. In reality, delay might be surprisingly short. Thus, the relative risk of CHD death declines by approximately 37% at 1 year after quitting smoking [48]. Furthermore, the surprisingly rapid CHD mortality reversals seen in Poland, Hungary and the Czech Republic [attributed to dietary changes] have recently been mirrored in communities adopting smoke-free legislation [49-51]. Thirdly, we considered only CHD deaths, not "competing causes" [52]. However, reductions in major risk factors would actually decrease deaths from other non-CHD causes, such as diabetes and common cancers [53]. Fourthly, the treatment uptakes in 2010 were assumed to remain constant at levels measured in 1999. In fact, more substantial improvements may be anticipated [18,54]. Our model estimates should therefore not be viewed as precise projections, but are usefully indicative of the changes in CHD mortality rates which might be expected under our three alternative scenarios.

## Conclusion

In conclusion, without specific interventions, a substantial increase in CHD deaths in Beijing is likely to occur. This will reflect worsening risk factors compounded by population ageing. However, modest, but eminently achievable, improvements in major risk factors could actually decrease the CHD burden. Future prevention strategies clearly need to be substantially strengthened. Furthermore, these results may be cautiously generalizable to other middle income countries addressing their own cardiovascular disease epidemics.

## Abbreviations

CHD: Coronary heart disease

## Competing interests

The authors declare that they have no competing interests.

## Authors' contributions

JC contributed to the conception and design, acquisition of data, analysis and interpretation of data, drafted and wrote the paper. DZ contributed to the conception and design, acquisition of data, analysis and interpretation of data, critical revision of this paper for important intellectual content, obtaining funding, administrative support and supervision. ZCZ contributed to the acquisition of data, critical revision of this paper for important intellectual content and technical support. JAC contributed to the conception and design, analysis and interpretation of data, critical revision of this paper for important intellectual content and supervision. JL contributed to the acquisition of data, critical revision of this paper for important intellectual content and technical support. WW contributed to the acquisition of data, critical revision of this paper for important intellectual content and technical support. JYS contributed to the acquisition of data, analysis and interpretation of data and technical support. SC contributed to the conception and design, obtaining funding and supervision, analysis and interpretation of data, drafting of this paper, and critical revision of this paper for important intellectual content.

All authors read and approved the final manuscript.

## Pre-publication history

The pre-publication history for this paper can be accessed here:



## Supplementary Material

Additional file 1 **Supplementary Appendix.** Calculation of CHD mortality resulting from change in a specific risk factor: β-coefficient and relative risk values. Click here for file
