# Supplementary material for: The impact of demographic and risk factor changes on coronary heart disease deaths in Beijing, 1999–2010
Source: BMC Public Health. 2009 Jan 22;9:30. doi: 10.1186/1471-2458-9-30 (PMC2637858; doi:10.1186/1471-2458-9-30)
Supplement: Additional file 1 — Supplementary Appendix. Calculation of CHD mortality resulting from change in a specific risk factor: β-coefficient and relative risk values. [file 1471-2458-9-30-S1.doc]

**Supplementary Appendix**

**Calculation of CHD mortality resulting from change in a specific risk factor: ß-coefficient and relative risk values**

| **Risk Factors** | ***ß-Coefficient **** | **Relative Risk †** | **Source** |
| --- | --- | --- | --- |
| **Cholesterol, mmol/L** | *0.65* | - | Meta-analyses of large cohort studies (Law et al., 1994; Law and Wald, 2002) ^1,2^ |
| **Smoking, %** | *-* | 1.60(1-9cig/d)  1.80(10-19cig/d)  2.10(>20cig/d) | Chinese cohort study (Chen et al., 1997; Yuan et al., 1996) ^3,4^ |
| **BMI, kg/m^2^** | *0.02* | - | Asia-Pacific region cohort study (Asia Pacific Cohort Studies Collaboration, 2004) ^5^ |
| **Diabetes, %** | *-* | 2.0 | Meta-analyses of large cohort studies (Nesto, 2001; Keen et al., 1999) ^6,7^ |
| **Diastolic blood pressure, mmHg** | *0.053* | - | Meta-analyses of large cohort studies (Lewington et al., 2002) ^8^ |

BMI indicates body mass index

***** ß-Coefficient shown for youngest age groups aged 35 to 44 years. Coefficients attenuated in older groups.

**†** Relative risk used to calculate population attributable risk in 1999 and again in 2010.

**References**

1. Law MR, Wald NJ, Thompson SG: **By how and how quickly does reduction in serum cholesterol concentration lower risk of ischaemic heart disease?** *BMJ* 1994, 308: 367-372

2. Law MR, Wald NJ: **Risk factor thresholds: their existence under scrutiny.** *BMJ* 2002, 324: 1570-1576

3. Chen ZM, Xu Z, Collins R, Li WX, Peto R: **Early health effects of the emerging tobacco epidemic in China.** *JAMA* 1997, 278: 1500-1504

4. Yuan JM, Ross RK, Wang XL, Gao YT, Henderson BE, Yu MC: **Morbidity and mortality in relation to cigarette smoking in Shanghai, China: a prospective male cohort study.** *JAMA* 1996, 275: 1646-1650

5. Asia Pacific Cohort Studies Collaboration: **Body mass index and cardiovascular disease in the Asia-Pacific Region: an overview of 33 cohorts involving 310 000 participants.** *Int. J. Epidemiol.* 2004, 33: 751-758

6. Nesto R.: **CHD: a major burden in type 2 diabetes.** *Acta Diabetol* 2001, 38: S3-S8

7. Keen H, Clark C, Laakso M: **Reducing the burden of diabetes: managing cardiovascular disease.** *Diabetes Metab Res Rev* 1999, 15: 186-196

8. Lewington S, Clarke R, Qizilbash N, Peto R, Collins R, for the Prospective Studies Collaboration: **Age-specific relevance of usual blood pressure to vascular mortality: a meta-analysis of individual data for one million adults in 61 prospective studies.** *Lancet* 2002, 360: 1903-1912
